# Association between low health literacy and adverse health behaviors in North Carolina, 2016

**DOI:** 10.13023/jah.0403.02

**Published:** 2023-01-01

**Authors:** Manan Roy, Adam Hege, Erin D. Bouldin

**Affiliations:** Appalachian State University, roym1@appstate.edu; Appalachian State University; Appalachian State University

**Keywords:** Appalachia, health literacy, omitted variable bias, treatment effect, health behavior, BRFSS

## Abstract

**Introduction:**

Health literacy (HL) is an urgent public health challenge facing the U.S. HL is a critical factor in health inequities and exacerbates underlying social determinants of health.

**Purpose:**

This study assesses the association between low HL (LHL) and adverse health behaviors, which contribute to poor health.

**Methods:**

Researchers used North Carolina’s 2016 Behavioral Risk Factor Surveillance System data, namely, the Health Literacy optional module which asks respondents to rate how difficult it is for them to get health-related advice or to understand medical information (verbal or written). Health behaviors analyzed were excessive alcohol consumption, lack of adequate exercise and sleep, and irregular medical and dental check-ups. The sample was divided into four age categories (18–49, 50–64, and 65–75, and 76 and older) for statistical comparisons. Stata 15 and a user-written Stata command, - *psacalc*-, were used to examine the relationships by addressing omitted variable bias in OLS regressions.

**Results:**

Findings indicate that LHL has a direct robust relationship with not exercising, inadequate sleep, irregular health and dental checkup, and health screenings across different age groups. Among women, LHL is associated with getting a Pap test in 3 years as opposed to more than 3 years.

**Implications:**

The adverse behaviors can explain the mechanisms underlying the link between LHL and adverse health outcomes. Further research on the causal relationship between LHL and adverse health behaviors using longitudinal data on a broader geographic region is warranted.

## INTRODUCTION

Health literacy (HL), an urgent public health challenge facing the U.S.,^1^,^2^ is one of the overarching goals of Healthy People 2030. The Healthy People framework defines HL along both personal and organizational aspects. Personal HL captures the extent to which individuals can find, understand, and use information and services to inform health-related decisions and actions for themselves and others. Organizational HL demonstrates the degree to which organizations *equitably enable* individuals to find, understand, and use information and services to inform health-related decisions and actions for themselves and others.^1^ Health literacy can play a vital role in addressing health equity and eliminating disparities to improve the health and well-being of all populations by assuring equitable access to programs and supports.^1^ However, more than one-third of American adults have limited HL, and just over 10% are considered proficient.^2^

The literature shows that low health literacy (LHL) can exacerbate underlying social determinants of health. For example, research has highlighted that due to LHL, many healthcare agencies are challenged at reaching citizens most in need, and this can lead to an underutilization of preventive services, misuse of medications, and an inability to provide basic health education.^3^,^4^ As a result, those with LHL have been found to be more likely to have poorer mental health outcomes and nutritional intakes and to be more likely to end up in an emergency room for basic healthcare needs.^3^,^4^ In addition, older adults and those in lower socioeconomic classes have tended to encounter the most significant impacts of LHL on health.^3^,^4^

Rurality and the underlying socioeconomic and accessibility challenges faced in many rural communities contributes significantly to health disparities.^5^ When examining rural health, much attention has been placed on the Appalachian region. In addition, the central and southcentral subregions of Appalachia have encountered the poorest health outcomes in recent years. The 2017 *Health Disparities in Appalachia* report shows that the Appalachian Region lags behind the nation in mortality rates for major chronic conditions, higher depression prevalence, and physically and mentally unhealthy days.^6^ Many of these disparities are rooted in social determinants, but the prevalence of adverse health behaviors, which could be linked with health literacy, cannot be overlooked.^7^,^8^ In fact, recent research has investigated health literacy in relation to environmental health, BMI, and self-care practices in Appalachian adults and school-aged children.^9^–^12^ School-aged children with lower HL reported significantly lower likelihood of health-promoting behaviors, such as adequate physical activity and sleep; greater frequency of risky health behaviors, such as consumption of sugar-sweetened beverages, junk food, and screen time; and had higher BMI percentiles and lower quality of life.^11^

The 29 western counties of North Carolina (NC) are in the southcentral Appalachian region, and much of the state is rural. A 2020 study^13^ in NC examined the associations between HL and healthcare access and several chronic health conditions (i.e., cancer, diabetes, and heart disease). However, the analysis did not explore the relationships between HL and health behaviors. To fill this gap, the current study specifically explored the relationship between LHL and adverse health behaviors in NC. It contributes to the literature by assessing a potential mechanism through which HL can influence health outcomes: LHL may increase adverse health behaviors (such as excessive alcohol consumption, irregular medical and dental check-ups, inadequate sleep, and inadequate exercise) which in turn adversely affect health. The other contribution of the study is methodological. It uses a novel omitted-variable-bias adjusted treatment effect estimator to assess the association between LHL and adverse health outcomes. This method overcomes the inability of ordinary least squares (OLS) estimation to address omitted variable bias.

## METHODS

### Data

The study used 2016 NC Behavioral Risk Factor Surveillance System (BRFSS) data. The BRFSS is a telephone survey of adults aged 18 and older who are not institutionalized. All data collected through the BRFSS are self-reported. The HL questions were introduced as an optional module in 2016 and were only used in NC in that year.^14^ The study sample includes 4,911 adults aged below 90 without missing information on age and sex. The literature shows HL varies significantly with age.^15^–^17^ Accordingly, the sample was divided into four age groups: 18–49 (n = 1,889), 50–64 (n = 1,509), 65–75 (n = 1,062), and 75–89 (n = 451). Stata 15 and a user-written command, -*psacalc*-, were used to examine the relationships.

### Measures

HL was measured by three questions: (1) “How difficult is it for you to get advice about health or medical topics if you need it?”; (2) “How difficult is it for you to understand information that doctors, nurses and other health professionals tell you?”; and (3) “You can find written information about health on the Internet, in newspapers and magazines, and in brochures in the doctor’s office and clinic. In general, how difficult is it for you to understand written health information?”. For each of the questions, response options included: “very easy,” “somewhat easy,” “somewhat difficult,” and “very difficult.” The not-applicable response category for the first question was, “I don’t look for health information” and for the third question was, “I don’t pay attention to written health information.” Following Rafferty et al.’s (2020) approach, these not-applicable responses were coded as missing for the analysis.^13^ “Somewhat difficult” and “very difficult” responses were combined into one category: “difficult.” The treatment variable, LHL, was defined as having difficulty with one or more of the three HL tasks.^13^ For outcomes, the study considered several binary measures (yes, no) of adverse health behavior:

1. not exercising outside of work in the past month
2. sleeping less than 6 hours, on average, in a 24-hour period
3. visiting a dentist/dental clinic within the past 5 years
4. binge drinking, defined as having five or more drinks (for males) or four or more drinks (for females) on one or more occasions in the past month
5. smoking every day
6. among women only, having a Pap test within the past 3 years

Several socioeconomic and demographic characteristics were included in the regression models. These included age and polynomials of age; categorical variables for male sex, white and non-Hispanic race/ethnicity, being married, being employed, educational attainment (less than high school, some college, college degree), annual household income (less than $25,000, less than $50,000, less than $75,000, greater than $75,000) and homeownership; and an interaction term for sex and age. Since the use of health services may be influenced by insurance coverage, the regression models for dental visits and Pap tests also include a variable indicating having any health insurance. All regression models also include county fixed effects to control for time-invariant county characteristics.

### Statistical Analysis

Empirical research using ordinary least squares regressions suffer from omitted variable bias (OVB)—whether they omit controls available in the data (observed characteristics) from the regression models or whether they cannot control for unobservable attributes that influence both the treatment variable and the outcomes that are not available in the data. Moreover, OLS and its non-linear counterparts assume that selection on observed characteristics (SOO) is random and that selection on unobserved characteristics (SOU) is zero. In the absence of a randomized experiment, neither of these assumptions hold true. Accordingly, the OLS estimates cannot be interpreted as causal effects.

Therefore, Oster^18^ proposes an OVB-adjusted estimator that allows the OVB to be proportional to coefficient movements scaled by the change in R^2^ (from the regressions) as the covariates are introduced going from the Uncontrolled Model (with one or two covariates) to the Controlled Model (with all relevant and available covariates). The OVB-adjusted estimator assesses the degree to which omitted unobservable factors might possibly explain away the observed adjusted relation between LHL and the outcomes. Underlying the test is the assumption that the bias from *observed* variables contains useful information regarding the bias from *unobserved* variables. It is essentially a selection on unobservables test on the robustness of the estimated coefficient from the Controlled Model. Appendix A describes the method in more detail. The method does not account for other forms of bias resulting from misclassification or recall.

The OLS estimated coefficient for LHL in the Uncontrolled Model must be statistically significant (at *p* ≤ 0.01, *p* ≤ 0.05, or at *p* ≤ 0.10) to apply the OVB method. The movements in both the estimated coefficient of LHL and R^2^ from the Uncontrolled Model to the Controlled Model illustrate selection on observed characteristics and most likely on unobserved attributes, too. In this study, potential selection on unobserved characteristics can be explained partly by the cross-sectional nature of the BRFSS, with no information about the respondents’ history of sleeping, employment status, health status, and past health behaviors that may affect behaviors at the time of the survey.

To apply this test, one must set up a maximum attainable value of R^2^ (from the OLS regressions), called R_max_, which measures the maximum variance explained by both observed and unobserved variables. However, since empirical evidence suggests that R_max_=1 is too conservative, Oster proposed to set R_max_=1.3R^2^, where R^2^ measures the variability explained by observed covariates (Controlled Model).

## RESULTS

### Summary Statistics

Participants between 65 and 75 years are least likely to have LHL (Table 1). In terms of demographic and socioeconomic characteristics, across all age groups, respondents with LHL are statistically more likely to be non-white, less educated, have lower income, and be uninsured. People with LHL are less likely to be homeowners. Across all age groups, those with LHL have statistically lower likelihood of getting adequate sleep and are more likely to have had routine dental checkups more than 5 years ago compared to those with greater HL. In the two youngest age groups, respondents with LHL were more likely to not exercise in the past month compared to those with greater HL. Women with LHL are less likely to have had Pap tests in the last 3 years compared to women with greater HL. Overall, unadjusted comparisons show that NC residents with LHL have more adverse circumstances and health outcomes.

### Bias-Adjusted Treatment Effect Results

In Table 2 none of the OLS estimates for the age category 65–75 were statistically significant and are not reported.^1^ Column 1 presents the OLS estimates for LHL, the standard errors, and R^2^ for the Uncontrolled Model, which additionally includes age and the male dummy only. Column 2 presents the same statistics for the Controlled Model. Following the caveat in Cinelli and Hazlett,^19^ only the estimated bounds on the bias-adjusted treatment effect are presented in Column 3.

Panel I presents the results for 18–49-year-old respondents. In Row A, the Uncontrolled Model estimates indicate individuals with LHL are 8.6% (*p* ≤ 0.05) more likely to get inadequate sleep on average. This estimate remains positive and statistically significant, and it drops to 5.9% (*p* ≤ 0.10) in Column 2 as more controls are added. The Oster test suggests in Column 3 that the relationship between inadequate sleep and LHL is robust at a level of R_max_=1.3R^2^ since bounds on the estimated LHL coefficient (5.9%, 6.3%) exclude zero. Similarly, Column 3, row C shows that the relationship between dental check-ups more than 5 years ago and LHL is robust at a level of R_max_=1.3R^2^ since bounds on the estimated LHL coefficient (4.9%, 5.7%) also exclude zero. Among women, LHL is associated with getting a Pap test within the past 3 years, as opposed to more than 3 years ago (Row D).

Panel II presents the results for 50–64-year-old respondents. The Uncontrolled Model estimates in Row A indicate individuals with LHL are 17.5% (*p* ≤ 0.01) more likely to get inadequate sleep on average. This estimate remains positive and statistically significant, and it drops to 11% (*p* ≤ 0.01) in Column 2 as more controls are included. The Oster test suggests in Column 3 that the relationship between inadequate sleep and LHL is robust at a level of R_max_=1.3R^2^ since bounds on the estimated LHL coefficient (11%, 16.4%) exclude zero. Similarly, in Row B, the Uncontrolled Model indicates individuals with LHL are 20.8% (*p* ≤ 0.01) more likely to not exercise in the past month. This estimate remains positive and statistically significant, and it drops to 10.6% (*p* ≤ 0.05) in Column 2 per the Controlled Model. Column 3 shows the estimated LHL coefficient in Column 2 is within the reported lower and upper bounds (10.6% and 16.8%). Thus, the selection on unobservables test provides evidence that reported results in Column 2 are robust.

Panel III presents the results for respondents who are at least 76 years old. In Row A, the Uncontrolled Model estimates indicate individuals with LHL are 16.9% (*p* ≤ 0.10) more likely to get inadequate sleep on average. This estimate remains positive and statistically significant, and it drops to 10.2% (*p* ≤ 0.10) in Column 2 as more controls are included. The Oster test suggests in Column 3 that the relationship between inadequate sleep and LHL is robust at a level of R_max_=1.3R^2^ since bounds on the estimated LHL coefficient (6.4%, 10.2%) exclude zero.

## IMPLICATIONS

LHL is a public health concern since it adversely affects a population's ability to follow and utilize necessary and often crucial health advice and recommended behavioral changes. The findings suggest that LHL is relatively common in NC— about 10–16% of community-dwelling adults across different age groups have LHL. The principal contribution of this study is in identifying the plausible mechanisms underlying the growing evidence on the link between LHL and adverse health conditions. The study finds LHL has a direct robust relationship with not exercising, inadequate sleep, irregular health and dental checkup, and health screenings across different age groups, corroborating existing literature on associations between LHL and the outcomes^3^,^4^,^13^ while strengthening the evidence by addressing OVB in the estimations.

These behaviors could explain the mechanisms underlying the link between LHL and adverse health outcomes, such as higher emergency-care use and increased hospitalization, worse self-management of chronic diseases, and higher rates of mortality.^3^,^4^ These robust estimates could explain the underlying mechanisms, since OVB is addressed after accounting for education levels that are often closely related to HL levels.

However, echoing Rafferty et al., the limitations of this study include self-reported data subject to measurement error and recall biases.^13^ Moreover, this study used cross-sectional data from NC and does not represent the experiences of other adults in the U.S. This method additionally assesses the sensitivity of the treatment effect to OVB and does not provide point estimates of the relationship. It also does not address other forms of bias, such as those arising from misclassification of health literacy status due to misreporting or recall error common in surveys.

The Calgary Charter on Health Literacy defined health literacy as the use of a wide range of skills that improve peoples’ ability to act on information to live healthier lives.^20^ These skills include reading, writing, listening, speaking, numeracy, critical analysis, as well as communication and interaction skills. It also noted that “*behavior change is a valid outcome of improved health literacy.”* The NC Institute of Medicine’s Task Force on Health Literacy provided 14 recommendations to raise and improve health literacy levels in the state.^21^ Further research on the causal relationship between LHL and adverse health behaviors using longitudinal data on a broader geographic region is warranted. For a more health literate and healthier society, policymakers can support interventions and programs to make health information available and accessible.

**SUMMARY BOX**

**What is already known about this topic?**

Low health literacy is a major public health issue across the United States and is linked to adverse health outcomes across the lifespan. However, there is limited research on the linkages between low health literacy and adverse health behaviors that subsequently result in adverse health outcomes, especially in rural states such as North Carolina.

**What is added by this report?**

This study sought to explore the relationship between low health literacy and adverse health behaviors. Findings indicate that low health literacy is associated with several adverse health behaviors such as inadequate exercise, inadequate sleep, and irregular medical, dental, and health screenings that adversely affect health.

**What are the implications for future research?**

Work remains to better explain the mechanisms that link LHL and adverse health outcomes. Further research is needed on the causal relationship between LHL and adverse health behaviors using longitudinal data on a broader geographic region.

## Supplementary Information



## Figures and Tables

**Table 1:** Summary statistics

Variable	Obs	Mean	SD*	Diff. in means	*p*-value	Obs	Mean	SD*	Diff. in means	*p*-value	Obs	Mean	SD*	Diff. in means	*p*-value	Obs	Mean	SD*	Diff. in means	*p*-value
	**18–49 years old**					**50–64 years old**					**65–75 years old**					**76 years and older**				
*** Treatment***																				
Low health literacy	1,889	0.122	0.327			1,509	0.138	0.345			1,062	0.106	0.309			451	0.159	0.366		
*** Health behavior outcomes***																				
No exercise (1= no physical activity past month)	1,889	0.169	0.375	0.099	0.000	1,509	0.250	0.433	0.209	0.000	1,062	0.284	0.451	0.007	0.872	451	0.408	0.492	0.098	0.123
Sleep < 6 (1 = sleep <6 hours)	1,889	0.129	0.335	0.087	0.000	1,509	0.133	0.340	0.176	0.000	1,062	0.083	0.277	0.08	0.003	451	0.112	0.315	0.157	0.000
Dental visit 5 (1 = dental visit within 5 years)	1,889	0.123	0.328	−0.02	0.398	1,509	0.114	0.318	0.105	0.000	1,062	0.078	0.269	0.109	0.000	451	0.066	0.249	0.059	0.065
Dental visit 5+ (1 = dental visit > 5 years ago)	1,889	0.110	0.313	0.114	0.000	1,509	0.125	0.330	0.138	0.000	1,062	0.139	0.346	0.087	0.011	451	0.204	0.403	0.173	0.001
Smoke everyday (1 = now smoke daily)	1,889	0.150	0.357	0.068	0.006	1,509	0.138	0.345	0.092	0.000	1,062	0.072	0.258	0.078	0.002	451	0.056	0.231	0.026	0.379
Binge drinker (1 = binge drinker)	1,889	0.180	0.385	0.052	0.056	1,509	0.106	0.308	−0.006	0.789	1,062	0.038	0.191	0.012	0.541	451	0.017	0.131	−0.021	0.221
Had a PAP test in the last 3 years (1 = Yes, females only)	1,889	0.370	0.483	−0.134	0.000	1,509	0.273	0.445	−0.072	0.030	1,062	0.025	0.157	0.001	0.928	451	0.000	0.000	0.000	.
*** Covariates***																				
*Demographics*																				
Male (1 = male)	1,889	0.468	0.499	0.032	0.360	1,509	0.466	0.499	−0.007	0.849	1,062	0.453	0.498	−0.001	0.988	451	0.407	0.492	0.115	0.070
Age (reported age in years)	1,889	33.741	9.283	0.343	0.600	1,509	56.706	4.395	−0.138	0.673	1,062	69.430	3.103	−0.217	0.482	451	80.478	3.619	0.387	0.407
White (1 = white)	1,889	0.585	0.493	−0.087	0.012	1,509	0.729	0.445	−0.089	0.007	1,062	0.803	0.398	−0.158	0.000	451	0.809	0.394	−0.064	0.209
Non-white (1 = non-white)	1,889	0.362	0.481	0.078	0.022	1,509	0.234	0.423	0.094	0.003	1,062	0.174	0.379	0.13	0.001	451	0.171	0.376	0.073	0.133
Married (1 = married)	1,889	0.508	0.500	−0.05	0.158	1,502	0.646	0.478	−0.183	0.000	1,061	0.650	0.477	−0.141	0.003	449	0.483	0.500	−0.068	0.288
*Education*																				
High school (1 = high school degree)	1,889	0.249	0.432	0.081	0.008	1,509	0.254	0.435	0.036	0.263	1,062	0.315	0.465	0.125	0.007	451	0.311	0.464	−0.099	0.099
Less than HS (1 = education < high school)	1,889	0.128	0.335	0.193	0.000	1,509	0.127	0.333	0.294	0.000	1,062	0.110	0.313	0.178	0.000	451	0.233	0.423	0.273	0.000
Some college (1 = some years in college)	1,889	0.370	0.483	−0.096	0.005	1,509	0.322	0.467	−0.068	0.050	1,062	0.327	0.469	−0.152	0.001	451	0.270	0.445	−0.062	0.283
College (1 = college degree)	1,889	0.251	0.434	−0.176	0.000	1,509	0.296	0.457	−0.26	0.000	1,062	0.247	0.431	−0.15	0.000	451	0.179	0.384	−0.105	0.033
*Economic factors*																				
Employed (1 = employed)	1,882	0.709	0.454	−0.129	0.000	1,501	0.595	0.491	−0.282	0.000	1,061	0.176	0.381	−0.046	0.227	450	0.026	0.160	−0.031	0.132
Health insurance (1 = insured)	1,880	0.806	0.396	−0.207	0.000	1,508	0.908	0.290	−0.104	0.000	1,059	0.990	0.101	−0.02	0.054	450	0.993	0.082	−0.006	0.597
Inc < $15,000 (1 = annual income<$15k)	1,889	0.083	0.276	0.066	0.001	1,509	0.091	0.288	0.135	0.000	1,062	0.050	0.219	0.066	0.002	451	0.115	0.319	0.183	0.000
Inc < $25,000 (1= annual income<$25k)	1,889	0.173	0.379	0.102	0.000	1,509	0.124	0.330	0.137	0.000	1,062	0.148	0.355	0.117	0.001	451	0.217	0.413	0.06	0.257
Inc < $50,000 (1 = annual income<$50k)	1,889	0.207	0.405	−0.011	0.693	1,509	0.184	0.388	0.038	0.186	1,062	0.245	0.430	−0.027	0.525	451	0.248	0.433	−0.101	0.069
Inc < $75,000 (1 = annual income<$75k)	1,889	0.132	0.339	−0.096	0.000	1,509	0.161	0.368	−0.147	0.000	1,062	0.148	0.356	−0.107	0.003	451	0.101	0.301	−0.085	0.028
Inc > $75,000 (1 = annual income>$75k)	1,889	0.244	0.430	−0.14	0.000	1,509	0.302	0.459	−0.192	0.000	1,062	0.190	0.392	−0.122	0.002	451	0.079	0.271	−0.02	0.574
Own home (1 = homeowner)	1,889	0.546	0.498	−0.118	0.001	1,509	0.828	0.378	−0.133	0.000	1,062	0.901	0.299	−0.101	0.001	451	0.906	0.292	0.029	0.449
*Regional characteristics*																				
Appalachian County (1 = Appalachian county)	1,889	1.819	0.385	0.025	0.363	1,509	1.826	0.379	−0.053	0.063	1,062	1.769	0.422	0.039	0.358	451	1.774	0.419	−0.028	0.608

NOTES:

* SD = standard deviation.

Bias-Adjusted Treatment Effect Results

**Table 2:** Low health literacy and adverse health behaviors by age group

Outcomes		(1) Uncontrolled Model	(2) Controlled Model	(3) Identified Set
**I. 18–49 years old**				
A. P(Sleep < 6 hours)				
Low Health Literacy	*Estimate*	0.0861†	0.0599*	[0.0599, 0.06280]
	*Robust S.E*.	(0.0352)	(0.0352)	
	*R* *^2^*	0.008	0.057	
	N	1889	1882	
B. P(Dental visit in past 5 years)				
Low Health Literacy	*Estimate*	−0.0199	−0.0551†	[−0.075, −0.055]
	*Robust S.E*.	(0.0245)	(0.0278)	
	*R* *^2^*	0.005	0.060	
	N	1,889	1,873	
C. P(Dental visit more than 5 years ago)				
Low Health Literacy	*Estimate*	0.112§	0.0568*	[0.04991, 0.0568]
	*Robust S.E*.	(0.0346)	(0.0319)	
	*R* *^2^*	0.022	0.109	
	*N*	1,889	1,873	
D. P(Pap in the last 3 years)				
Low Health Literacy	*Estimate*	−0.224§	−0.160§	[−0.160, −0.023]
	*Robust S.E*.	(0.0560)	(0.0522)	
	*R* *^2^*	0.071	0.304	
	*N*	1,044	1,040	
E. P(Binge drinking)				
Low Health Literacy	*Estimate*	0.0504	0.0722†	[0.0722, 0.07424]
	*Robust S.E*.	(0.0369)	(0.0368)	
	*R* *^2^*	0.022	0.097	
	N	1,889	1,882	
**II. 50–64 years old**				
A. P(Sleep < 6 hours)				
Low Health Literacy	*Estimate*	0.175§	0.110§	[0.110, 0.16383]
	*Robust S.E*.	(0.0434)	(0.0414)	
	*R* *^2^*	0.032	0.127	
	N	1,509	1,497	
B. P(No exercise)				
Low Health Literacy	*Estimate*	0.208§	0.106†	[0.106, 0.16873]
	*Robust S.E*.	(0.0467)	(0.0476)	
	*R* *^2^*	0.028	0.131	
	N	1,509	1,497	
**III. 76 years and older**				
A. P(Sleep < 6 hours)				
Low Health Literacy	*Estimate*	0.169*	0.102*	[0.06482, 0.102]
	*Robust S.E*.	(0.0866)	(0.0592)	
	*R* *^2^*	0.064	0.273	
	N	451	448	

NOTES:

All analyses are weighted using BRFSS survey weights. Statistical significance:

* *p* < 0.01

† *p* < 0.05

§ *p* < 0.1

Model estimates are generated via user-written Stata command *-psacalc-*. Robust standard errors are included in parentheses in Columns (1) and (2).

## Footnotes

1 The results are available upon request.
