# Supplementary material for: Association between low health literacy and adverse health behaviors in North Carolina, 2016
Source: J Appalach Health. 2023 Jan 1;4(3):23–38. doi: 10.13023/jah.0403.02 (PMC10655731; doi:10.13023/jah.0403.02)
Supplement: Supplementary file 1 [file Roy_Additional_File.docx]

**Appendix A: Further Details on Method**

The Oster approach^18^ begins with a model with some observed confounders and some unobserved confounders. Specifically, consider the following regression model:

*Y* = β*LHL* + γω^0^ + *W*_2_ + ε*;*

where *Y* is the outcome of interest, *LHL* is the LHL indicator, and ω^0^ is a vector of observed control variables ω^0^_1_, ..., ω^0^*_J_:* The index *W*_2_ is not observed. We define *W*_1_ = γω^0^ and assume all elements in ω^0^ are orthogonal to *W*_2_, so *W*_1_ and *W*_2_ are orthogonal.

We then define the proportional selection relationship between the observed characteristics and unobserved attributes as follows:

$$\delta\frac{\sigma_{1LHL}}{\sigma_{1}^{2}}=\frac{\sigma_{2LHL}}{\sigma_{2}^{2}},$$

where $\sigma_{iLHL}=cov\left( W_{i}, LHL \right), \sigma_{i}^{2}=var\left( W_{i}^{2} \right), i=1,2$and $\delta$ is the coefficient of proportionality. Since we do not make any assumptions about $\delta$ now, this relationship always holds for some $\delta$.

Let the coefficient on *LHL* from the uncontrolled regression of *Y* on *LHL* be β_0_ and the R-squared from that regression be *R*_0_. Let the coefficient from the controlled regression of *Y* on *LHL* and ω^0^ be β_1_ and the R-squared *R*_1_. Finally, let *R*_max_ be the R-squared from a hypothetical regression of *Y* on *LHL*, ω^0^, and *W*_2_ (full model).

The omitted variable bias-adjusted treatment effect can be approximated as follows:

$\beta^{*}\approx\beta_{1}-\delta[$β_0_ $- \beta_{1}]\frac{R_{max}-R_{1}}{R_{1}-R_{0}}.$

Oster suggests two ways to assess the robustness of coefficient stability using the above bias-adjusted estimator:

One approach is to calculate the value of $\delta$ (the coefficient of proportionality) for which β = 0 by assuming a value for *R*_max_. That is, the degree of SOU relative to SOO that would be needed to explain away the estimated effect under exogeneity.

The second approach is to set bounds on *R*_max_ and $\delta$ to develop a set of bounds for β. Following the partial identification literature, the estimator can be denoted as β* (*R*_max_*,*$\delta$ ). Now, without any additional assumptions, *R*_max_ is bounded between *R*_1_ (the R-squared in the controlled regression) and 1. Assuming $\delta$ *>* 0, that is the covariance between *LHL* and observed characteristics is of the same sign as the correlation between *LHL* and the unobserved covariates, bounds δ between 0 and an arbitrary upper bound δ.

The bounding set for $\beta$ is:

$$\Delta_{s}=\left[ \beta_{1}, \beta^{*}\left( \bar{R_{max}},1 \right) \right].$$

If the bounds of the set are outside the confidence interval on β₁ then the conclusions based on the controlled regression's coefficient is not robust. That is, if the identified set includes zero, the estimated treatment effect from the controlled regression is not robust. Finally, Oster uses empirical evidence from randomized control trial studies to determine a plausible value for $R_{max}: R_{max}=1.3*R_{1}$

In other words, if the identified set [$\beta_{1,}\beta^{*}(\left\{ 1.3*R_{1},1 \right\},1)]$excludes zero or the estimated which produces $\beta$ = 0 with *R*_max_ = 1*:*3 *R*_1_ exceeds 1, then we can conclude the estimated coefficient is robust. The cut-off value of $\delta$ = 1 is appropriate since it suggests the observables are at least as important as the unobservable attributes. This is because researchers typically choose regression controls which they believe ex ante are the most important.^18^

[Reference 18, from full manuscript: Oster E. Unobservable selection and coefficient stability: Theory and evidence. *J Bus Econ Stat* 2017;37(2):187–204. DOI: [10.1080/07350015.2016.1227711](https://doi.org/10.1080/07350015.2016.1227711)]
